# Hospitals and Vivisection

**Published:** 1898-01-29

**Authors:** 


### The Hospital

A Journal of

The Medical Sciences and Hospital Administration.

Vol. XXIII.—No. 592.] Edited by Sir Henry Burdett, K.C.B., and Solomon C. Smith, M.D., M.R.C.P. [Jan. 29, 1898.

### Hospitals and Vivisection.

A correspondence has been published between the Lord Mayor, as president of the Hospital Sunday Fund for London, and Mr. Sydney G. Trist, secretary of the London Anti-Vivisection Society, on the ruling of the Lord Mayor that, at the annual general meeting of the Fund, a proposed reference by the Rev. J. F. B. Tinling to the subject of vivisection in hospitals was out of order and could not be permitted. Mr. Trist was directed by his committee to express their regret that the Lord Mayor should have stifled discussion on what they deemed to be legitimate subject of debate; and he enters into various details with regard to occurrences which Mr. Tinling intended to bring into prominence. The word "correspondence" is, perhaps, scarcely a correct description of what has occurred, the letters of the Lord Mayor, or rather of his secretary, having been limited to bare acknowledgments of those received; while Mr. Trist, on the part of his committee, has been more or less descriptive and argumentative. The committee are of opinion that his Lordship could not be aware of the connection between animal experimentation in physiological and bacteriological laboratories and human experimentation in some of the leading hospitals; and they quote a truly lamentable case, in which a patient in a London hospital was directed to eat half a pound of butter in the course of a day, not in order that he might be fattened as material for a cannibal feast to be given by some member of the staff, but solely for the purpose of "testing" some question which was in dispute. Our readers will remember that something similar was done by Mr. Pickwick, who took an extra glass of punch in order to ascertain whether it contained lemon-peel, which always disagreed with him, and who, comforted by the absence of the noxious agent in question, took several more glasses afterwards. Not very long ago, a terrible indictment was brought by an anti-vivisection society against a physician for having permitted certain experimental administrations of drugs in his hospital—administrations which had been carefully watched by students, colleagues, and other miscreants of like kind, and the results of which had been published in a medical journal. In that instance, the society had hopelessly written itself down an ass before it was disclosed that the person who took the drugs was the physician himself. In the case now in question, we are told that the patient had a "biliary fistula"; and we may safely conclude that, if he were a person of ordinary intelligence and ordinary humanity, he would be perfectly willing to eat half a pound of butter if, by this not very terrible martyrdom, he could enable his physician to determine some question with regard to the influence of a liberal supply of fat upon the functions of the liver.

We know nothing whatever of Mr. "Trist," beyond the unhappy suggestiveness of his name; but the average secretary of any "anti" society is a person who has to earn his living by suppressing as much truth, and by inventing as much fiction, as the interests of his particular contention may require. Even so, we think there should be limits to his activity; and, if this be once granted, it is not too much to say that hospitals should be secured against the practices of his particular form of industry. Experimentation on living animals, and the practical work of hospitals, are so seldom carried on by the same persons that even those who disapprove of the former need not on that account feel any scruples about supporting the latter. They would probably admit that knowledge, even when it had been gained by experiment, should be utilised rather than wasted, and they would probably be vexed with any hospital surgeon who, being called in to themselves in an emergency, said, 'I could easily save your life or relieve your suffering, but, unfortunately, the methods of procedure calculated to accomplish either purpose have been ascertained by experiment, and my conscience will not permit me to apply them." And, moreover, the folly of an outcry against "human experimentation" in hospitals depends upon the obvious consideration that, without experiment, there could be no improvement. If someone had not "experimented" with ligatures, surgeons would still be searing with hot iron the stumps left after amputation. The continuance of experiment is necessary to the welfare of the human race, and the only antecedent condition should be the possession of adequate knowledge by the experimenter. Everything fresh which is made known is calculated to suggest something fresh which may be done, or which, under proper precautions, may be attempted; and this is legitimate experimentation, whether it be upon human creatures or upon the lower animals. When hyperpyrexia was found to be a source of imminent danger, it was an experiment to immerse the patient in a cold bath; but it was an experiment springing from knowledge, and, destined to be crowned by success. With great submission to Mr. Trist and his friends, there are very few of us who would not gladly be made the subjects of analogous experiment, if we were placed under conditions of analogous gravity.

